# Development of a Novel Convolution to Interactive Capture and Recalibration Enhancement Module for Underwater Fish Detection in Sensor Networks

**DOI:** 10.3390/s26134290

**Published:** 2026-07-06

**Authors:** Vinie Lee Silva-Alvarado, Ali Ahmad, Sandra Sendra, Jaime Lloret

**Affiliations:** Instituto de Investigación para la Gestión Integrada de Zonas Costeras, Universitat Politècnica de València, C/Paranimf, 1, Grao de Gandia, 46730 Valencia, Spain

**Keywords:** object detection, attention mechanism, YOLO, smart aquaculture, underwater detection, aquatic monitoring, deep learning

## Abstract

Underwater optical sensor networks are essential for fish monitoring, yet imagery is often affected by illumination variability, low contrast, and complex backgrounds. Attention mechanisms are vital for feature representation in deep networks, yet existing approaches often struggle with spatial information loss and limited multi-scale interaction under such challenging conditions. This paper introduces Convolution to Interactive Capture and Recalibration Enhancement (C2ICARE), a lightweight attention module designed to overcome these challenges. The principal contribution of C2ICARE is the adaptation of memory interaction principles into an edge-oriented attention framework that enhances feature discrimination while maintaining computational efficiency. The architecture employs three core innovations: a 1:3 memory-feature split to preserve context while reducing cost, parallel multi-scale depthwise convolutions (3 × 3 and 7 × 7) for fine-grained and broad feature extraction, and a cross-branch interaction mechanism coupled with a ConvNeXt-style feed-forward network that avoids dimensionality reduction. Experimental results on an underwater fish dataset demonstrate that YOLO26n with C2ICARE achieves a mean average precision (mAP@0.5:0.95) of 0.7033, outperforming Coordinate Attention (+3.8%), FasterBlock (+1.7%), and CBAM (+0.4%) while adding only 0.05M parameters and 0.16 GFLOPs. Multi-objective Pareto Frontier analysis confirms that C2ICARE provides an effective balance between accuracy, efficiency, and generalization for resource-constrained deployment. EigenCAM visualizations further validate that the model focuses on biological morphology rather than background noise. Its lightweight design enables seamless integration with underwater sensor networks and fog platforms for real-time fish detection in aquaculture, commercial fisheries, and scientific research. Future work will investigate broader marine applications and cross-platform deployment scenarios. The code is available on GitHub.

## 1. Introduction

Underwater sensor networks have become essential infrastructure for marine ecosystem monitoring, fisheries management, intelligent aquaculture, and autonomous underwater exploration [1,2,3]. These networks typically integrate acoustic, environmental, and optical sensors deployed on stationary observatories, autonomous underwater vehicles, or floating platforms to collect continuous data from the marine environment. Among these sensing modalities, optical cameras provide the richest source of information for species identification, abundance estimation, and behavioral analysis [4]. However, underwater visual perception remains particularly challenging due to light attenuation, scattering effects, low image quality, complex backgrounds, dense object distributions, and the frequent presence of small targets, all of which can substantially degrade detection performance and real-time reliability [5,6,7]. Consequently, advanced deep learning (DL) models have been widely adopted to enhance feature representation and robustness in such sensor-driven environments. Among these, attention mechanisms have emerged as a fundamental paradigm inspired by the selective perception capability of the human visual system, enabling neural networks to emphasize informative features while suppressing irrelevant information [8]. Furthermore, modern attention formulations, including self-attention, facilitate the modeling of long-range dependencies and complex feature interactions, thereby improving representation learning in challenging visual scenarios [9,10].

Attention mechanisms have recently emerged as the cornerstone of modern computer vision, enabling models to prioritize task-relevant features over noisy backgrounds [11,12]. By recalibrating spatial and channel-wise information, these modules allow deep neural networks to achieve unprecedented precision in complex environments [13,14,15]. However, underwater fish detection remains a formidable challenge. The inherent optical properties of water, such as light absorption and scattering, result in low contrast, turbidity, and variable lighting that, combined with high intra-class variability, often degrade the performance of standard architectures [16,17]. 

In order to mitigate these issues, various attention strategies have been proposed. The Squeeze-and-Excitation (SE) [18] network pioneered channel-wise recalibration but at the cost of discarding spatial positional data. Subsequent developments, such as the Convolutional Block Attention Module (CBAM) [19], introduced spatial branches but relied on aggressive dimensionality reduction, which often leads to the loss of fine-grained morphological details. Coordinate Attention (CoordAtt) [20] and its derivative, Improved Coordinate Attention (ImCA) [21], attempted to embed positional information through parallel pooling. Recent alternatives like FasterBlock and Partial Convolution (PConv) [22] succeed in reducing FLOPs but lack explicit multi-scale spatial interaction, often leaving fine-grained features underexplored. Nevertheless, these modules remain limited by their lack of explicit multi-scale spatial modeling, often failing to capture the intricate structures of small or partially occluded aquatic species.

The pursuit of more robust attention has led to specialized architectures in other domains, though often at the cost of increased complexity. For instance, in continual learning scenarios, the FALCON [23] approach utilizes contrastive attention to manage catastrophic forgetting, yet its dual-clustering loss adds significant training overhead. In neuromorphic computing, the Spiking Transformer (STAtten) [24] integrates spatial-temporal attention to handle spike-based data efficiently; however, its block-wise computation strategy, while energy-efficient, is specifically optimized for event-based sensors rather than standard RGB frames. Similarly, SEEN-DA [25] uses semantic entropy to guide attention in domain adaptation, which is effective for cross-domain stability but requires complex inter-domain attention branches that increase latency.

More recently, the CARE Transformer [26] introduced a decoupled design philosophy, separating features into memory and detail streams to preserve long-range dependencies [27]. While achieving impressive results in mobile-friendly classification, its reliance on dynamic memory units and asymmetric dual interactions creates a computational bottleneck unsuitable for the dense, real-time prediction required underwater sensor platforms and autonomous underwater robotics, particularly on resource-constrained edge and fog devices where memory bandwidth and power consumption are severely limited [28]. 

These limitations motivate the need for a lightweight, interactive design tailored for embedded underwater sensor applications [29,30]. In this work, we propose C2ICARE (Convolution to Interactive Capture and Recalibration Enhancement). Inspired by the decoupled philosophy of CARE, C2ICARE replaces expensive attention layers with a hybrid architecture of 3 × 3 and 7 × 7 multi-scale depthwise convolutions and a single cross-branch projection [31,32]. The module is designed to operate directly on the image sensor data stream, performing efficient multi-scale feature recalibration to improve detection reliability under variable environmental conditions. Its lightweight architecture makes it suitable for edge and fog computing platforms that are co-located with imaging sensors in underwater observatories, aquaculture facilities, and fishing vessels.

This module was validated using an underwater fish dataset specifically because it represents a high-entropy environment where traditional feature extractors fail due to flickering light and overlapping targets [33,34]. Furthermore, to bridge the gap between laboratory results and real-world deployment in sensor networks, we introduce a multi-objective selection framework based on Pareto Frontier analysis [35]. This approach identifies the most efficient architecture by balancing detection accuracy, inference speed, and parameter economy. Once the optimal model is selected, we perform a threshold optimization to solve the critical trade-off between precision and counting discrepancy [36]. This dual-stage strategy significantly reduces systematic bias, ensuring that the model provides reliable abundance estimates for biological surveys rather than just high-mAP scores.

Collectively, the existing literature reveals a critical research gap. Although contemporary attention mechanisms improve feature representation, they typically achieve these gains through additional attention branches, dynamic memory updates, or complex interaction pathways, thereby increasing computational overhead and limiting their suitability for real-time deployment on resource-constrained underwater sensor platforms. Furthermore, current architectures rarely address the simultaneous requirements of multi-scale feature extraction, contextual information preservation, and computational efficiency, all of which are essential for reliable underwater object detection in sensor networks. Consequently, the central objective of this work is not merely to introduce another attention module, but to develop an architecture that explicitly balances representational capability with deployment efficiency on sensor-equipped fog devices. Guided by this objective, C2ICARE adopts a one-to-three memory feature partitioning strategy to preserve contextual information with minimal overhead, employs parallel 3 × 3 and 7 × 7 depthwise convolutions to enhance multi-scale feature modeling, and incorporates a lightweight cross-branch recalibration mechanism to strengthen feature interaction without the complexity of conventional attention operations. In doing so, C2ICARE directly addresses the principal limitations of existing attention frameworks while remaining suitable for embedded underwater vision systems integrated into sensor networks for aquaculture, fisheries monitoring, and scientific research.

The main contributions of this work are organized as follows:

i. Design and implementation of C2ICARE module, a novel lightweight attention mechanism featuring a one-to-three memory-feature split and a feed-forward recalibration network without channel reduction.
ii. Integration of C2ICARE into existing YOLO architectures as a drop-in replacement for standard blocks through a wrapper module.
iii. Validation of the developed module on an underwater fish dataset using a three-phase progressive freezing strategy and expanded uncertainty with k equals two, following international measurement standards.
iv. Development of a multi-objective selection framework based on Pareto Frontier analysis to identify the most efficient architecture for deployment on resource-constrained sensor platforms.
v. Proposal of a wireless sensor network architecture for the deployment of the C2ICARE module in fish farms.

The rest of this paper is organized as follows: Section 2 describes the proposed C2ICARE module; Section 3 presents the materials and methods, including the sensor network deployment architecture; Section 4 reports the experimental results; Section 5 provides the discussion and limitations; and finally, Section 6 draws the conclusions.

## 2. Proposed C2ICARE Module

This section establishes the theoretical foundations and structural progression leading to the proposed lightweight operator. First, we outline the fundamental architecture of the deCoupled duAl-interactive lineaR attEntion (CARE) mechanism [26]. Finally, we introduce the formal mathematical framework and structural components of the proposed Convolution to Interactive Capture and Recalibration Enhancement (C2ICARE) module, demonstrating how it resolves these limitations through a purely convolutional design.

### 2.1. CARE Block Architecture

Within this framework, the CARE Block serves as the fundamental processing unit. This component integrates three key architectural pillars: a token mixer responsible for segregating data flows into memory and detail branches, a dynamic memory unit that preserves critical information across network stages, and a dual interaction module that coordinates communication between local and global features. This structure allows the block to maintain a rich feature representation while optimizing resource utilization.

### 2.2. Implementation and Architectural Limitations of the CARE Block

While the CARE block [26] offers a compelling decoupled design, its integration into real-time object detection frameworks on hardware-constrained edge and fog devices presents significant implementation hurdles.

Although the original architecture achieves high performance on resource-rich platforms with dedicated computing power, its reliance on multi-branch parallel tensor operations and custom memory-unit updates generates frequent memory access operations, inherently increasing latency during continuous inference on constrained hardware.

Furthermore, the logic of the original block is closely tied to its initial classification-heavy design, creating a black box effect that complicates its portability into dense prediction tasks like those found in the YOLO family. The computational overhead of managing multi-branch projections (Q, K, V) and specialized activations often results in a rigid implementation that lacks the “plug-and-play” versatility required for embedded marine systems. Crucially, these complex memory mechanisms and non-standard operations induce high memory bandwidth demands, severely restricting deployment on edge and fog devices with limited RAM and processing capacity. These integration barriers motivated our proposal: C2ICARE. By distilling the decoupled philosophy into a purely convolutional operator, we eliminate the structural rigidity of the original block, providing a lightweight, hardware-friendly alternative that can be seamlessly integrated into diverse detection architectures without compromising real-time performance [37].

### 2.3. C2ICARE Module Overview

**Figure 1:**
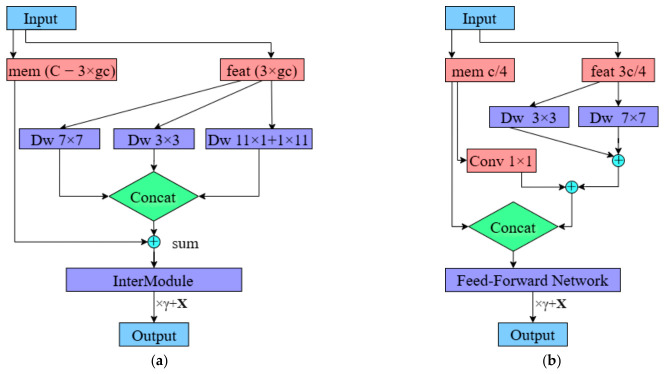
Architectural comparison between (**a**) the CARE Block and (**b**) the proposed C2ICARE Block.

The C2ICARE (Convolution to Interactive Capture and Recalibration Enhancement) module was designed as a lightweight yet powerful computational unit to augment the representational capacity of object detection models. Let $X\in R^{C\times H\times W}$ denote the input feature tensor, where C, H, and W represent the number of channels, height, and width, respectively. The module transforms X into an enriched output tensor Y of identical dimensions through a series of decoupled transformations (Figure 1). The C2ICARE Block replaces the complex three-branch parallel processing of CARE with a simplified memory-feature split, multi-scale depthwise convolutions, a single cross-branch projection, and a lightweight ConvNeXt FFN.

In order to empirically validate this strategic reduction in architectural overhead and quantify the efficiency gains achieved by this streamlined design, a rigorous comparative computational complexity analysis was conducted against the baseline CARE block. Both blocks were evaluated under identical tensor environments with an input feature map dimension of 256 × 20 × 20. The theoretical computational cost was quantified using the standard metrics of total learnable parameters and Floating Point Operations (FLOPs), calculated via the tracking utilities of the ptflops framework, defining FLOPs as twice the number of multiply accumulate operations (MACs) to account for both multiplication and addition steps within each computational cycle.

As detailed in Table 1, the baseline CARE block configurations configured with the operational multi-head attention mechanism (if_att = True, mem_dim = 64, att_head_dim = 16) demand 852,672 parameters and exhibit a computational overhead of 0.686 GFLOPs. Conversely, the proposed integrated C2ICARE module, enclosed within its channel-splitting and recombination wrapper structure, significantly reduces the structural scale to 206,816 parameters and curtails the execution intensity down to 0.166 GFLOPs. This contrast is primarily attributed to the inherent design philosophy of the original CARE block, which is structurally oriented toward high-capacity server-side deep learning architectures that prioritize maximum feature expression over resource-constrained computational efficiency.

### 2.4. C2ICARE Block Architecture

The internal structure of the C2ICARE block operationalizes the decoupled interaction mechanism through a sequential, multi-stage pipeline. Designed to bypass the intensive memory and computational bottlenecks of traditional attention layers on edge and fog hardware, the block isolates and channels feature data into parallel processing streams. This workflow minimizes memory access operations while preserving multiscale context. The formal mathematical formulation of this sequence is detailed below.

Given the input tensor X, we apply a channel-wise partition operator to decompose the feature space into two functionally distinct subspaces (Equation (1)):

(1) $$X_{mem},X_{feat}=\mathrm{Split}\left(X,\alpha \right)$$

where $\alpha \in \left(0,1\right)$ is the memory ratio. The subspaces are defined such that $X_{mem}\in R^{C_{m}\times H\times W}$ and $X_{feat}\in R^{C_{f}\times H\times W}$, with $C_{m}=\left\lfloor C\cdot \alpha \right\rfloor$ h and $C_{f}=C-C_{m}$. In our implementation, an empirical ratio of α = 0.25 was adopted to balance structural context preservation with detailed feature extraction.

In order to capture spatial dependencies across multiple receptive fields while maintaining computational efficiency, the feature branch $X_{feat}$ undergoes parallel depthwise convolution operations. Let $K_{k}$ denote a depthwise convolution operator with a kernel of size k ×k. The transformation is expressed in Equation (2):

(2) $$X_{feat}^{\prime }=K_{3}\left(X_{feat}\right)+K_{7}\left(X_{feat}\right)$$

By utilizing element-wise summation of 3 ×3 and 7 ×7 kernels, the module extracts both fine-grained morphological details and broader semantic context without the parameter overhead of standard dense convolutions.

In order to facilitate information exchange between the decoupled branches, the memory branch is projected into the feature manifold via a pointwise convolution operator $P_{m\to f}:R^{C_{m}}\to R^{C_{f}}$ (Equation (3)):

(3) $$X_{fused}=X_{feat}^{\prime }+P_{m\to f}\left(X_{mem}\right)$$

Following this fusion, the branches are recombined via concatenation along the channel dimension, $U=\left[X_{mem};X_{fused}\right]\in R^{C\times H\times W}$. This tensor is then processed by a Feed-Forward Network (FFN) based on the ConvNeXt paradigm, defined by the composite function in Equation (4):

(4) $$F\left(U\right)=P_{2}\left(\sigma \left(P_{1}\left(\mathrm{BN}\left(U\right)\right)\right)\right)$$

where BN denotes Batch Normalization, $P_{1}$ and $P_{2}$ are 1 ×1 convolutions with an expansion factor of 2, and σ represents the Gaussian Error Linear Unit (GELU) activation function. The final output Y is governed by a residual mapping with learnable layer scaling (Equation (5)):

(5) $$Y=X+\mathrm{diag}\left(\gamma \right)\cdot F\left(U\right)$$

where $\gamma \in R^{C}$ is a parameter vector initialized at 10^−6^, allowing the network to adaptively calibrate the contribution of the block during the optimization process.

### 2.5. C2ICARE Wrapper Module

**Figure 2:**
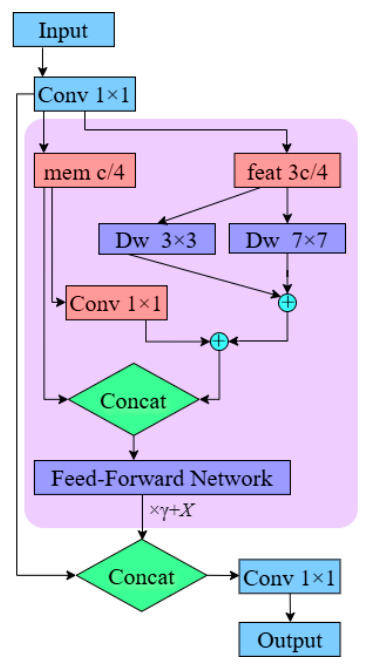
Architecture of the C2ICARE wrapper module.

In order to ensure seamless integration into YOLO-family architectures, the block is encapsulated within a residual-style wrapper module (Figure 2), where the purple-shaded region corresponds to the internal C2ICARE Block. The process begins with the application of an expansion convolution $P_{exp}$ that projects the input tensor $X_{in}\in R^{C_{1}\times H\times W}$ into a higher-dimensional space $2\cdot C_{c}$, where $C_{c}=\left\lfloor C_{1}\cdot e\right\rfloor$ and e is the expansion factor. The resulting tensor is split equally into two branches, $a,b\in R^{C_{c}\times H\times W}$. While branch a acts as an identity shortcut to preserve original information, branch b undergoes a sequence of n C2ICARE blocks, denoted by the composite operator $B^{n}$. Finally, both branches are concatenated and projected back to the original space via a reduction convolution $P_{red}$, producing the final output $Y_{out}=P_{red}\left(\left[a;B^{n}\left(b\right)\right]\right)$. This design enables the network to learn residual corrections over an identity base, optimizing gradient flow.

## 3. Materials and Methods

This section describes the experimental framework designed to validate the proposed C2ICARE module. A standardized training pipeline is introduced, comprising a three-phase progressive freezing strategy, a robust data augmentation protocol, a multi-objective evaluation framework, and an analytical threshold optimization method for abundance estimation.

### 3.1. Fish Dataset

The experimental evaluation was conducted using the Fish_YOLO_Dataset [38], a specialized corpus totaling 5.4 GB structured in a standard ZIP format optimized for YOLO-family architectures. In order to ensure comprehensive validation under realistic marine conditions, the dataset features high environmental variability, including two distinct lighting profiles captured across different oceanographic campaigns and highly variable water turbidity levels. The dataset partition consists of 5652 images for Training, 309 images for Validation, and 918 images for Testing, and is targeted at automated counting, abundance measurement, and fish stock assessment applications. The annotations encompass four primary biological categories, with the training split containing a dense distribution of 19,107 localized targets: 4424 instances of Bluewhiting, 5116 of Herring, 4800 of Mackerel, and 4767 belonging to the Mesopelagic group (Table 2).

### 3.2. Proposed Evaluation and Training Protocol

In order to ensure a fair and reproducible comparison between different neural components, a standardized training pipeline characterized by controlled gradient flow and specific data augmentation is proposed.

#### 3.2.1. Three-Phase Progressive Freezing Trainer

To establish an equitable evaluation framework under transfer learning conditions, a specialized protocol based on three-phase progressive freezing was implemented. This strategy ensures that the observed performance gains are strictly attributable to the proposed architectural modifications rather than stochastic variations in pre-trained weights. Furthermore, it directly addresses the feature interference problem that typically occurs when integrating randomly initialized modules into a network with established weights. By managing the gradient flow across three distinct stages, the protocol facilitates a smooth adaptation of the data manifold while preserving the spatial hierarchy learned during pre-training.

During the initial phase of feature recalibration (epochs 1–15), training is strictly confined to the module under test at layer 10 and the detection head at layer 23. By freezing the remainder of the architecture, the new parameters are forced to learn transformation functions that are compatible with the existing feature representations. For this stage, a learning rate of 1 × 10^−3^ and a weight decay of 5 × 10^−4^ provide sufficient mobility for the new weights while preventing premature overfitting to the training textures.

As the training progresses into the multi-scale integration phase (epochs 16–30), the feature pyramid network (layers 11-22) is additionally activated. The purpose of this transition is to allow the network to learn the correct propagation of the enhanced features across various detection scales. While the backbone remains frozen to maintain foundational stability, the optimization of the neck ensures that multi-scale aggregation adapts to the new data flow dynamics. At this juncture, a Cosine Annealing scheduler is introduced to smooth the convergence toward a more stable local minimum.

The process culminates in a global fine-tuning stage (epochs 31–50) involving a complete unfreezing of all layers. In this final period, the weight decay is adjusted to 1 × 10^−4^ and the backbone learning rate is set to a conservative 5 × 10^−3^. This configuration allows for subtle global adjustments, optimizing the model for the specific textures of the target environment without compromising the generalization capabilities inherited from pre-training. All comparative analyses are conducted under this deterministic scheme using fixed seeds 0, 1 and 2, ensuring that every architectural variant is evaluated under identical boundary conditions.

#### 3.2.2. Robust Data Augmentation and Regularization

**Figure 3:**
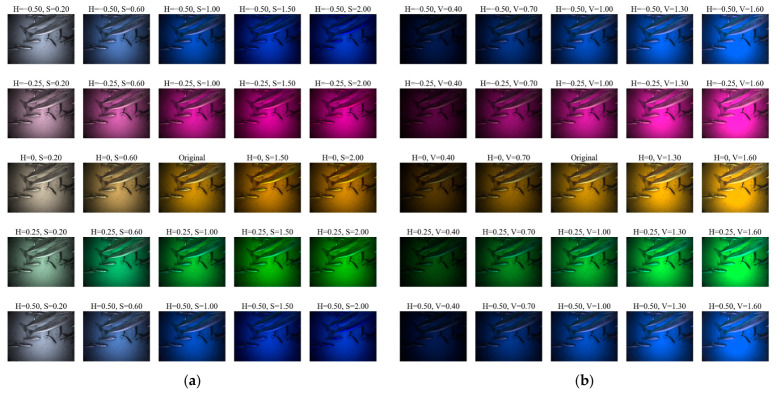
Graphical analysis of the hyperparameter space for the HSV-based subaquatic data augmentation strategy: (**a**) multi-spectral simulation matrix mapping continuous hue shifts (±0.5) against saturation multipliers (from 0 to 2.0) at a constant value baseline (V = 1.0); and (**b**) illumination and backscattering simulation matrix mapping continuous hue shifts against value multipliers (from 0.4 to 1.6) at a constant saturation baseline (S = 1.0). The center of each grid corresponds to the unaugmented baseline image.

In order to ensure that the proposed modules maintain high performance under varying environmental conditions, a robust data augmentation and regularization strategy was implemented with the primary objective of decoupling detection features from highly variable underwater backgrounds while ensuring robustness against fluctuations in luminosity and contrast [39]. In order to achieve color and lighting invariance, HSV shifts, comprising hue ± 0.5, saturation multipliers from 0 to 2, and value multipliers from 0.4 to 1.6, were applied to simulate the non-uniform light absorption and spectral shifts characteristic of different water columns (Figure 3). 

This approach effectively forces the model to prioritize morphological and structural features over inconsistent color cues that often degrade in aquatic environments. Furthermore, background decoupling and occlusion robustness were addressed through a combination of mixup (0.1), cutmix (0.1), and copy-paste (0.2) techniques. These methods prevent the model from overfitting to specific benthic backgrounds and enhance its ability to detect partially occluded targets by creating synthetic scenes where objects appear in novel spatial contexts with varying levels of foreground-background interference. In order to supplement these augmentations, a dropout rate of 0.1 was applied to the newly integrated modules to mitigate the risk of neuronal co-adaptation, while an early stopping mechanism with a patience of 5 epochs was implemented to prevent overtraining based on validation loss monitoring. Finally, to ensure the scientific reliability of the results, all reported values are expressed as mean ± expanded uncertainty (k = 2), providing a 95% confidence interval in strict accordance with the Guide to the Expression of Uncertainty in Measurement (GUM).

### 3.3. Multi-Objective Evaluation Framework

Reliable deployment of deep learning models in resource-constrained underwater environments requires simultaneous consideration of predictive accuracy, computational efficiency, and hardware feasibility. Consequently, model selection cannot be adequately guided by a single performance indicator, particularly when improvements in one objective are often accompanied by deterioration in another. Recent advances in multi-objective optimization have highlighted that optimal solutions should be evaluated from a Pareto perspective, where competing objectives are jointly considered to identify the most balanced and practically viable configurations [40,41].

Although conventional metrics, including mAP, GFLOPs, and parameter count, provide valuable insights into detection performance and computational complexity, they characterize individual attributes in isolation. As a result, these metrics do not fully capture the cumulative operational burden imposed on edge devices during continuous inference. More specifically, marginal gains in detection accuracy may incur disproportionate increases in computational cost, memory requirements, or processing latency, thereby reducing real-time deployability despite improved predictive performance.

Accordingly, the proposed framework introduces the Latency Performance Score (LPS), Generalized Efficiency Sparsity Index (GESI), and Parallel Efficiency Index (PEI) as derived composite indicators that consolidate complementary performance dimensions into interpretable decision variables. Rather than replacing standard evaluation metrics, these indices extend their utility by explicitly quantifying deployment-relevant trade-offs that remain obscured when accuracy and efficiency metrics are examined independently. Furthermore, the proposed formulation enhances the discriminatory power of the evaluation process by enabling holistic comparisons among competing architectures. Collectively, these derived indices establish a unified multi-objective assessment framework that supports Pareto-informed model selection and facilitates identification of architectures achieving an effective balance between detection capability and computational efficiency under practical deployment constraints.

Another evaluated metric was the Latency Performance Score (LPS). LPS evaluates the real-time viability of a model by relating its mean Average Precision (mAP@0.5:0.95) to its inference latency ($T_{inf}$), measured in milliseconds (Equation (6)). This metric effectively quantifies the accuracy-per-millisecond return, ensuring that models which achieve high precision at the cost of excessive delay are penalized. A higher LPS indicates a more responsive architecture suitable for high-frequency control loops, which can be used in many scenarios like in autonomous underwater vehicles (AUVs) [42].

(6) $$LPS=mAP@0.5:0.95/T_{inf}$$

The GFLOPs Efficiency Score Index (GESI) measures the algorithmic efficiency of the architecture. By normalizing the mAP@0.5:0.95 against the total GFLOPs, this index identifies models that utilize floating-point operations most effectively (Equation (7)). GESI is a crucial indicator for determining how well an attention module optimizes the feature extraction process without unnecessarily increasing the total computational workload.

(7) GESI=mAP@0.5:0.95/GFLOPs

In order to assess the structural compactness of the proposed modifications, the Parameter Efficiency Index (PEI) is utilized. This index represents the ratio of precision to the total number of parameters (P × 10^6^). A high PEI highlights architectures that achieve superior feature representation with a minimal memory footprint, which is essential for embedded systems with limited storage and cache capacity.

(8) $$PEI=mAP@0.5:0.95/\left(P\times 10^{6}\right)$$

Likewise, the Custom Score (S) serves as a unified decision-making metric by consolidating accuracy and efficiency into a single value. It is calculated as the arithmetic mean of the normalized F1-Score, LPS, PEI, and GESI (Equation (9)). The F1-Score is specifically included to ensure that the model maintains balanced precision and recall across all detection categories, preventing the selection of architectures that might be biased toward majority classes at the expense of rare but critical targets. By using normalized values, the framework ensures that each dimension contributes equally to the final ranking, regardless of its original units, facilitating a balanced comparison between highly accurate but heavy models and efficient but lightweight variants.

(9) $$S=\left(F{1-Score}_{norm}+LPS_{norm}+PEI_{norm}+GESI_{norm}\right)/4$$

Another metric was the Overfitting Score (Ω), which is introduced to quantify the generalization health of the training process. It is defined as the relative difference between the average validation loss (${\bar{L}}_{val}$) and training loss (${\bar{L}}_{train}$) over the final k epochs (where k = 5). Values close to 0 signify robust generalization, while positive values indicate overfitting. Conversely, negative values may suggest the presence of strong regularization, techniques implemented to encourage the learning of generalizable patterns rather than data memorization, or a comparatively simpler validation set.

(10) $$\Omega =\left({\bar{L}}_{val}^{k}-{\bar{L}}_{train}^{k}\right)/{\bar{L}}_{train}^{k}$$

The final model selection is governed by the Pareto Frontier Analysis, which identifies models that are non-dominated across multiple objectives. In this context, a model $M^{\prime }$ is considered superior to a model M if it simultaneously provides a higher Custom Score (S) and a lower Overfitting Score (Ω). This dual-constraint approach ensures that the chosen architecture is not only the most efficient but also the most stable across different data distributions (Equation (11)).

(11) $$P=S\left(M^{\prime }\right)\geq S\left(M\right)\wedge \Omega \left(M^{\prime }\right)\leq \Omega \left(M\right)$$

### 3.4. Analytical Threshold Optimization Strategy 

A formal method to minimize abundance estimation bias in counting tasks is proposed. By modeling the detection discrepancy $\Delta \left(\theta \right)=N_{pred}\left(\theta \right)-N_{gt}$ as a function of the confidence threshold θ, we can analytically derive the optimal operating point $\theta_{opt}$ such that $\Delta \left(\theta_{opt}\right)\to 0$.

In underwater automated quantification, selecting standard, static confidence thresholds (θ = 0.25 or θ = 0.50) introduces systematic counting errors due to the non-linear distribution of environmental noise, occlusion, and turbidity. In order to establish an objective criterion, this framework evaluates the continuous behavior of $\Delta \left(\theta \right)$ across the validation dataset. The discrete discrepancy values obtained at varying threshold increments are modeled using a continuous quadratic function. This empirical function maps the statistical equilibrium point where false positives and false negatives cancel out. Consequently, $\theta_{opt}$ is mathematically defined as the root of the optimization polynomial, providing a standardized baseline for operational fish counting that eliminates empirical trial-and-error configurations.

### 3.5. Proposed Wireless Sensor Network in Aquaculture

**Figure 4:**
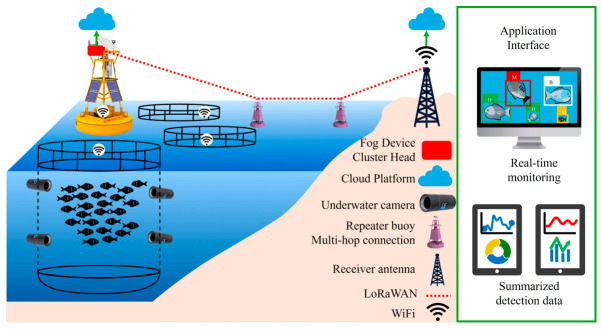
Hierarchical network architecture for fog-based fish detection in aquaculture, illustrating data flow from underwater cameras through Fog Computing to cloud platforms.

Aquaculture facilities require continuous, non-intrusive monitoring of fish welfare, biomass estimation, and environmental parameters. The proposed network architecture is hybrid (Figure 4), combining underwater cameras, environmental sensors, and wireless connectivity, and leverages buoy-based repeater networks to extend communication range in offshore installations [43].

At the underwater acquisition layer, the system employs low-cost, high-resolution cameras that output raw video for processing by the C2ICARE module. A cost-effective solution is the OE17-110 IP Subsea Camera (Imenco AS, Aksdal, Norway), specifically designed for aquaculture and caged fish farming applications, featuring a 1/2.7″ CMOS sensor and 1080p HD resolution. These cameras are connected via underwater Ethernet cables to a surface buoy that houses the processing infrastructure.

The processing architecture follows a Fog Computing paradigm: a floating platform, the surface buoy, contains a fog device, a Raspberry Pi 5 with AI HAT+, which executes the C2ICARE-enhanced detection model on the raw video streams. A single Raspberry Pi 5 with AI HAT+ can handle up to six parallel video streams [35], making it suitable for multi-cage operations where multiple cameras are deployed across different pens or cages. However, to scale monitoring across dozens of cages, the system adopts a time-division multiplexing strategy. The Fog Device sequentially polls each cage, processing the video streams from its cameras for a configurable duration, typically a few minutes, before moving to the next cage. Since the raw video streams are captured and stored without requiring real-time AI inference, the accelerator is only engaged during the active processing windows. When no detection is requested, the fog device simply relays the raw video to storage or performs lightweight compression. This approach enables a single Raspberry Pi 5 with AI HAT+ to monitor an entire farm with dozens of cages, as the computational load is distributed over time and the accelerator is used only when analytical results are needed.

**Figure 5:**
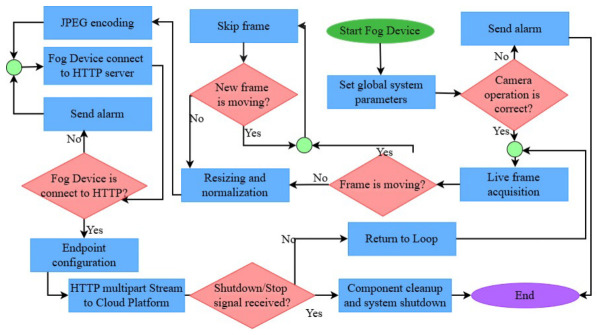
Operational flowchart of Fog Computing Device for real-time monitoring of fish farm.

For connectivity, the buoy is equipped with a Starlink maritime terminal as the primary communication link, providing high-bandwidth, low-latency connectivity to the cloud. The system transmits both the processed video streams with overlaid bounding boxes and classifications, and the unprocessed raw video from each camera (Figure 5). This dual-transmission strategy serves two critical purposes: it allows continuous monitoring and validation of the model’s inference performance, and it provides a valuable repository of raw, unannotated data that can be leveraged for future model retraining, fine-tuning, and domain adaptation under evolving environmental conditions. Additionally, statistical data such as counts, species distribution, behavioral alerts, and environmental parameters measured by auxiliary sensors are transmitted.

In the event of Starlink failure due to weather, satellite coverage gaps, or technical issues, the system falls back to a LoRaWAN-based backup link. In this mode, only summarized detection data, such as counts per species, critical alerts, and extreme environmental deviations, are transmitted, as LoRaWAN’s bandwidth is limited to a few kilobits per second. To extend the range of LoRaWAN communication, a network of repeater buoys is deployed between the farm and the coast, relaying the low-bandwidth data packets until they reach a coastal LoRaWAN gateway. From the gateway, the data is forwarded to the cloud via a wired broadband connection. This dual-link strategy ensures continuous monitoring even under connectivity disruptions.

## 4. Results

This section presents a comprehensive evaluation of the proposed C2ICARE module. First, the training dynamics are analyzed to validate the three-phase protocol. Second, a comparative analysis against state-of-the-art attention mechanisms is performed. Finally, a multi-objective evaluation and an analytical threshold optimization are conducted to ensure operational reliability. All experiments were implemented using the PyTorch v2.9.1 framework and conducted on a single NVIDIA GeForce RTX 3060 Laptop GPU with 16 GB of memory, paired with an Intel Core i7-12650H processor.

### 4.1. Training Dynamics and Ablation Analysis Results

**Figure 6:**
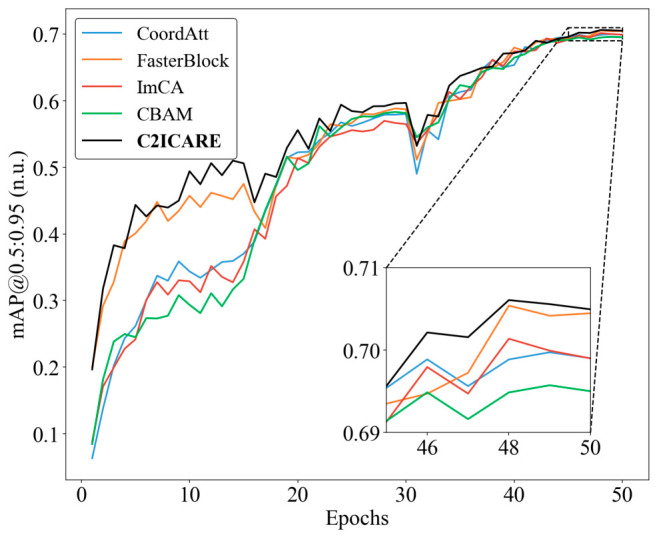
Mean Average Precision (mAP@0.5:0.95), expressed in non-dimensional units (n.u.), as a function of 50 training epochs.

The training evolution of the evaluated attention modules over 50 epochs, averaged across three independent runs with random seeds 0, 1, and 2, is illustrated in Figure 6. Transfer learning accelerates network convergence [44,45,46]. It avoids training models from scratch. The results represent the average of three independent runs (random seeds 0, 1, and 2). The numerical results from the training logs provide a granular validation of the three-phase progressive freezing protocol and the adaptability of each architectural variant.

During phase 1 (epochs 1–15), which focused on feature recalibration with a frozen backbone, the C2ICARE module demonstrated superior initial convergence. Starting from epoch 2, C2ICARE achieved an mAP@0.5:0.95 of 0.3175, significantly outperforming CBAM (0.1813) and ImCA (0.1708). By the end of this phase (epoch 15), C2ICARE reached a precision of 0.5059, maintaining a clear lead over the other variants and proving that the module can effectively extract meaningful features even when global network updates are restricted.

The transition to Phase 2 (epochs 16–30), characterized by the activation of the feature pyramid (neck layers), initially induced a temporary stabilization in all models as they adjusted to the new gradient flow. However, the recovery was rapid; by epoch 19, C2ICARE regained momentum with an mAP@0.5:0.95 of 0.5291, consistently staying ahead of the baseline variants. This phase confirmed that fine-tuning the neck is critical for multi-scale integration, with C2ICARE reaching 0.5966 by epoch 30, whereas ImCA and CoordAtt lagged at 0.5648 and 0.5799, respectively. 

In Phase 3 (epochs 31–50), where the entire architecture was unfrozen for global fine-tuning, the performance gap further widened. Although a brief drop in accuracy occurred at epoch 31 due to the global weight update, C2ICARE showed the most robust recovery, breaking the 0.60 threshold by epoch 34 (0.6225). From epoch 46 onward, C2ICARE maintained a consistent position above 0.70, reaching its peak at epoch 48 with a mAP of 0.7061. Compared to FasterBlock, which also showed strong performance at the end (0.7045), C2ICARE demonstrated a more stable ascent with fewer oscillations. These results confirm that the proposed module not only achieves the highest absolute precision but also provides a more reliable training trajectory for marine species identification tasks.

The integration of the C2ICARE module into the YOLO26n baseline yielded a significant performance increment while maintaining a lightweight computational profile. As shown in Table 3, C2ICARE achieved an mAP@0.5:0.95 of 0.7033 ± 0.0207. The results indicate that C2ICARE effectively enhances spatial-channel synergy. Despite a slight increase in parameters compared to CBAM.

### 4.2. Multi-Objective Evaluation Results

**Figure 7:**
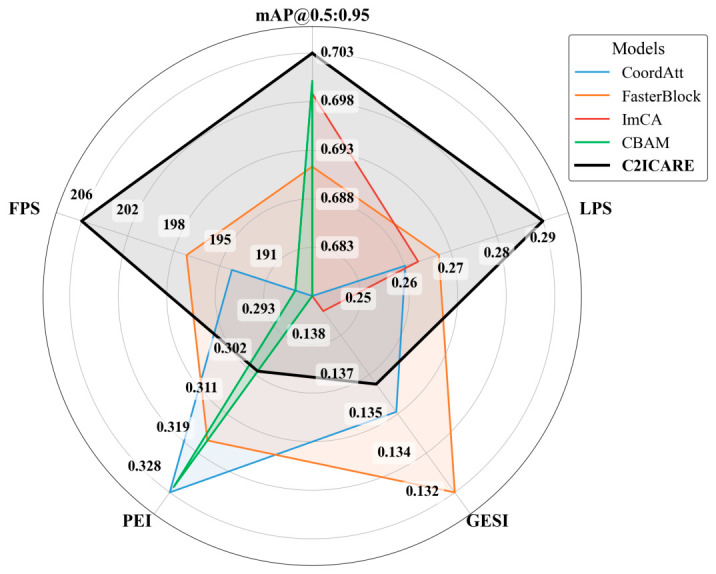
Radar chart comparing YOLO variants with different modules in layer 10. The GFLOPs axis is inverted; peripheral placement reflects lower computational demand and higher efficiency.

In order to assess operational efficiency, we utilized the Multi-Objective Evaluation Framework. As illustrated in the radar chart (Figure 7), the proposed C2ICARE module achieves the most robust balance across all efficiency metrics, outperforming both the baseline and other attention-based variants. This is further confirmed by the results in Table 4, where C2ICARE achieved the highest Custom Score (0.6189) and was identified as a non-dominated solution on the Pareto frontier. The negative Ω values across the top-performing models suggest strong regularization, indicating that the models are learning generalizable patterns rather than memorizing the complex underwater backgrounds.

### 4.3. Analytical Threshold Optimization Results

The application of the optimization strategy to the fine-tuned YOLO26n + C2ICARE model revealed a clear monotonic relationship between the confidence threshold and the counting discrepancy. As shown in Table 5, the total number of predicted individuals decreased from 3553 at θ = 0.10 to 1653 at θ = 0.60, relative to the constant ground-truth ($N_{gt}$ = 2366). The system reached its highest counting precision at θ = 0.35, yielding a minimum MAE of 0.421 ± 1.521 and a maximum per-image counting coefficient $R_{count}^{2}$ of 0.8806. Notably, the bias transitioned from overestimation to underestimation between θ = 0.30 and θ = 0.35, suggesting that the true optimal operating point lay within this interval.

**Figure 8:**
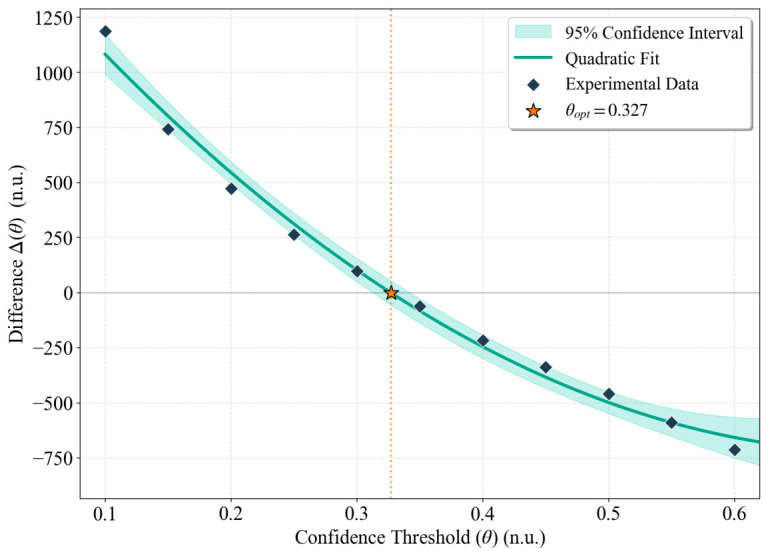
Quadratic regression of the detection discrepancy $\Delta \left(\theta \right)$ between predicted and ground-truth fish counts as a function of the confidence threshold θ.

In order to identify the precise threshold that minimizes systematic error, a quadratic regression was performed on the experimental data (Figure 8). Blue squares represent the experimental data. Experimentally, at this threshold, the model yields Δ = +1 (indicated by the yellow star point).

The resulting empirical model is defined by Equation (12):

(12) $$\Delta \left(\theta \right)=4725.4080\theta^{2}-6786.3310\theta +1713.8546$$

with $R_{fit}^{2}$ = 0.9913 indicating an excellent fit. Solving for $\Delta \left(\theta \right)\to 0$ yielded an analytical optimal threshold of $\theta_{opt}$ = 0.327. Experimental validation at this specific point produced a near-zero bias of 0.001 ± 1.715 fish per frame and a total discrepancy of only +1 fish across the entire test set.

Under these conditions, the model achieved an $R_{count}^{2}$ of 0.8846 and an MAE of 0.419 ± 1.494. While the MAPE remained relatively high (15.48% ± 59.58%), this is attributed to the presence of images with low fish density and partial occlusions, where a single misclassification results in a disproportionate relative error. Consequently, the low absolute bias and MAE confirm that the YOLO26n + C2ICARE model, operating at the analytically derived $\theta_{opt}$, provides a robust and reliable solution for biological abundance estimation in challenging underwater scenarios.

### 4.4. XAI Visualization

**Figure 9:**
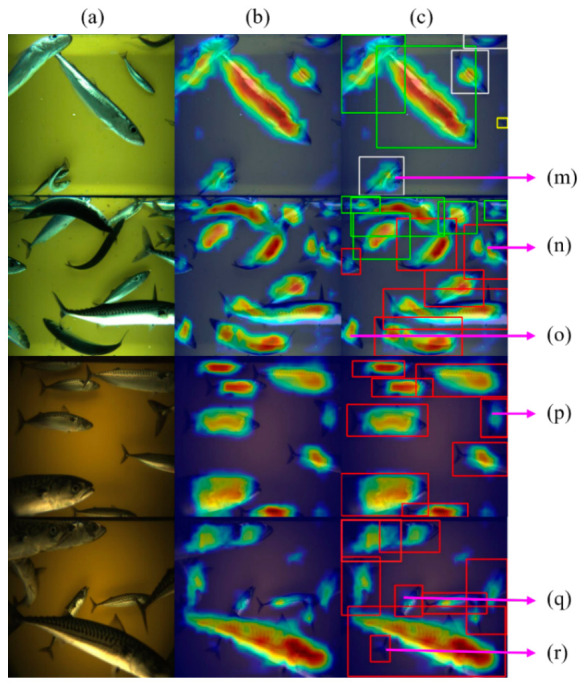
Visual explanation of the feature extraction via EigenCAM for the YOLO26n + C2ICARE model. The multi-class detection is color-coded as follows: mackerel (red), herring (green), bluewhiting (white), and mesopelagic (yellow). Column (**a**): raw frames; column (**b**): activation maps showing structural salience; column (**c**): overlap between heatmaps and final bounding box predictions. Cases (m)–(r) illustrate specific detection instances: (m) multiple individuals within a single bounding box with correct internal count; (n) fragmented detections due to occlusion; (o) activation heatmap without corresponding detection; (p) species-specific feature recognition under low light; (q)–(r) occlusion-driven fragmentation with accurate taxonomic classification.

In order to validate that the model focuses on fish morphology rather than background artifacts, we performed EigenCAM analysis. As shown in Figure 9, the activation maps for the YOLO26n + C2ICARE model exhibit precise spatial alignment with fish morphology. The heatmaps in column (b) demonstrate that structural salience is concentrated on target bodies, effectively filtering out complex benthic textures even in low-contrast or partially occluded scenarios. The correlation in column (c) confirms that bounding box predictions stem directly from these high-salience regions. 

A detailed examination of specific detection instances reveals the model’s high sensitivity and its current limitations in complex scenarios. In case (m), a single bounding box apparently encompasses two individuals; however, the internal count in this frame correctly identifies four herring (white). Conversely, case (n) displays three distinct heatmaps that are grouped into a single detection, likely due to edge or fog detection challenges when only caudal fins are visible within the receptive field.

The robustness of the analytical threshold ($\theta_{opt}$ = 0.327) is evidenced in case (o), where an activation map exists without a corresponding bounding box, indicating that the feature salience did not meet the required confidence for a formal detection. In terms of pattern recognition, case (p) demonstrates the model’s capacity to identify species-specific features, such as caudal morphology, even under extreme low-light conditions at the frame boundaries. Finally, cases (q) and (r) illustrate a common challenge in underwater computer vision: occlusion-driven fragmentation. When a fish passes in front of another, the visual continuity is severed, causing the model to generate two distinct bounding boxes for a single individual. Despite this fragmentation, the taxonomic classification remains accurate, highlighting the stability of the feature extraction regardless of partial body visibility.

### 4.5. Comparison Between C2ICARE and Baseline YOLO26n

**Figure 10:**
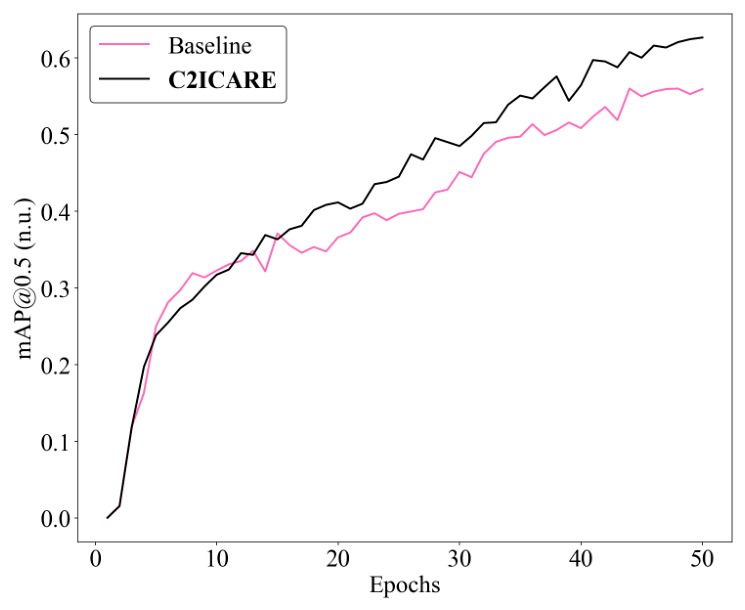
Training from scratch metrics for C2ICARE and baseline.

In order to rigorously evaluate the native feature extraction capacity of the proposed architecture, both models were trained completely from scratch without using any pre-trained weights (see Figure 10).

During the first ten epochs, the performance metrics remain highly similar due to the randomized parameter initialization inherent to training from scratch. However, a clear turning point occurs at epoch fifteen, where the C2ICARE model permanently outperforms the vanilla baseline. This performance gap widens steadily throughout the remaining iterations. By epoch 50, the proposed model reaches a mAP@0.5 of 0.626, whereas the unmodified baseline stalls at 0.559. Consequently, even in the complete absence of pre-trained weights, C2ICARE delivers a 6.7% absolute accuracy gain.

## 5. Discussion and Limitations

The experimental results demonstrate that C2ICARE consistently improves detection accuracy across YOLO architectures with minimal computational overhead. By replacing global pooling with a memory-feature split and large-kernel spatial attention with efficient multi-scale depthwise convolutions, the module captures complex spatial patterns while preserving positional information. This synergy makes it particularly suitable for underwater environments where occlusions and variable lighting are prevalent. However, the findings of this study are bounded by several contextual factors and methodological constraints that warrant further analysis.

Although C2ICARE is conceptually derived from CARE, both architectures were developed to address different deployment requirements. Whereas CARE emphasizes rich memory interaction mechanisms suitable for computationally capable platforms, C2ICARE was specifically designed for resource-constrained edge environments through lightweight memory feature partitioning and efficient feed-forward recalibration. Consequently, the novelty of C2ICARE lies not only in architectural modification but also in the deliberate adaptation of memory interaction principles for real-time embedded deployment. Nevertheless, a systematic benchmark between CARE and C2ICARE across diverse hardware platforms, incorporating latency, memory consumption, energy efficiency, and detection performance, remains an important direction for future investigation.

### 5.1. Analytical Threshold and Systematic Bias

A critical limitation arises in the Analytical Threshold Optimization. While the derived $\theta_{opt}$ = 0.327 achieved a near-zero discrepancy (Δ = +1) over the entire dataset, this result must be interpreted with caution. In scenarios of extreme low luminosity, some individuals may fail to reach the detection threshold entirely [47,48]. If the model fails to detect these real individuals but the global count remains balanced, the methodology might be overestimating the counts in other frames to compensate for these misses. Consequently, the apparent zero-bias could mask a combination of false negatives (due to visibility) and false positives, rather than reflecting perfect detection accuracy.

### 5.2. Structural and Architectural Constraints

The development of the C2ICARE module adopted the structural foundation of the original CARE block, utilizing a fixed configuration of DW 3 × 3 and DW 7 × 7 convolutions. While effective, this study did not perform a systematic ablation of alternative kernel sizes, and the current arrangement may not be the optimal hierarchy for all underwater feature scales [32,49]. Furthermore, the experiments were limited to 50 epochs. Although the performance hierarchy among the modules remained stable, it cannot be guaranteed that this trend will persist over longer training cycles.

### 5.3. Domain Specificity of Data Augmentation

The robust data augmentation strategy was specifically designed to decouple detection from the spectral shifts in the water column caused by various artificial lighting conditions [50,51]. While this approach proved successful in aquatic environments, where morphological features are more reliable than chromatic ones, the protocol is highly domain-dependent.

In fields such as precision agriculture, for instance, the detection of fruits or pests often depends strictly on specific color maturation stages and precise chromatic features [52]. In such contexts, the aggressive color jittering used in this study could be detrimental, as it would destroy the very color cues necessary for classification. 

Consequently, the generalizability of this work is strictly oriented toward aquatic environments, and its application to terrestrial domains is not recommended without a fundamental reassessment of the augmentation logic. The current pipeline, including its hyperparameter configuration and training schedule, was tailored to the specific constraints of underwater imaging; therefore, any transition to terrestrial datasets would require a significant recalibration to ensure optimal convergence under different physical conditions.

### 5.4. Technical and Statistical Constraints

Finally, several technical constraints persist that define the scope of this study. First, experiments were conducted on a single Northeast Atlantic dataset; generalization to other marine ecosystems or alternative architectures remains to be tested [53]. Regarding statistical stability, while three random seeds provided a stable indication of performance, a larger number of independent runs would yield tighter and more robust confidence intervals. Additionally, the module’s deployment on edge and fog devices was not characterized; thus, power consumption and memory bandwidth constraints may affect real-time performance on embedded robotic platforms [54]. Lastly, the 1:3 memory-feature split was determined empirically, suggesting that this configuration may require further tuning to adapt to different network depths or dataset complexities. Furthermore, while C2ICARE was validated within a single-stage YOLO architecture for marine targets, its architectural transferability to multi-stage detectors or broader object detection scenarios involving non-rigid corporate structures remains an open challenge. Deploying this module in alternative underwater environments, such as high-turbidity tropical environments or shallow coral reefs, will also introduce distinct scattering phenomena that could alter the spatial activation patterns of the attention mechanism, necessitating dynamic kernel-size selection policies.

### 5.5. Potential Applications and Future Perspectives

Beyond the improvements observed in underwater fish detection, the proposed C2ICARE module could potentially provide a generic lightweight attention mechanism for a wide range of resource-constrained computer vision applications. Because the module enhances feature discrimination while introducing only a marginal computational overhead, it is likely to be particularly suitable for fog-AI scenarios in which memory capacity, inference latency, and energy consumption represent major deployment constraints.

In intelligent aquaculture, the proposed framework could support continuous, non-invasive monitoring of fish populations through autonomous estimation of abundance, species composition, behavioral changes, and welfare indicators. When integrated with the WSN architecture described in this work, C2ICARE might enable scalable real-time monitoring across multiple cages using low-cost embedded hardware, thereby reducing manual inspection requirements while improving the temporal resolution of biological observations. The simultaneous transmission and archival of raw imagery could additionally facilitate continual model refinement through incremental learning, domain adaptation, or active annotation strategies as environmental conditions evolve.

The architectural principles underlying C2ICARE are not inherently limited to underwater imagery. Since the proposed attention mechanism primarily operates by improving the interaction between local morphological information and broader contextual representations, it could potentially benefit numerous dense-object detection problems characterized by complex backgrounds, partial occlusions, illumination variability, or small object sizes. Consequently, the module may be applicable to precision aquaculture, wildlife monitoring, marine ecology, environmental surveillance, autonomous robotic inspection, industrial quality control, intelligent transportation systems, and medical image analysis, where accurate feature localization under computational constraints remains essential.

Furthermore, the lightweight design of C2ICARE could facilitate deployment on heterogeneous fog-computing platforms, including embedded GPUs, NPUs, FPGAs, and low-power AI accelerators. Such flexibility may enable its incorporation into distributed IoT and Fog Computing infrastructures, where local inference is preferred to minimize communication latency and bandwidth requirements while preserving scalability.

Although these prospective applications remain to be experimentally validated, the present findings suggest that the proposed module represents a promising general-purpose attention operator that could be readily integrated into existing one-stage and potentially two-stage detection architectures with minimal architectural modifications. Future investigations should therefore evaluate its robustness across diverse visual domains, detection backbones, and hardware platforms to establish its broader generalization capability under heterogeneous real-world operating conditions.

## 6. Conclusions

In this paper, we have introduced C2ICARE, a novel and efficient attention mechanism designed to enhance feature representation in underwater fish detection. Our module addresses the limitations of standard attention methods by replacing conventional global pooling with a memory-feature split and substituting large-kernel spatial attention with multi-scale depthwise convolutions. This architectural design allows the network to capture long-range dependencies while preserving precise positional and morphological information, which is critical for identifying species under variable lighting and high-density conditions. Furthermore, we have proposed a wireless sensor network architecture for the practical deployment of C2ICARE, integrating underwater cameras, fog computing devices, and hybrid satellite-LoRaWAN connectivity in fish farms.

Extensive experiments across various YOLO architectures demonstrate the effectiveness of our approach, where C2ICARE consistently outperformed established baselines like CoordAtt, FasterBlock, ImCA and CBAM. Beyond detection accuracy, we investigated the reliability of the model for abundance estimation, providing a systematic framework for analytical threshold optimization. Our findings show that an optimal threshold can minimize systematic bias to near-zero levels, addressing a fundamental requirement for measurement-oriented biological applications. Furthermore, EigenCAM visualizations confirmed that the module induces the network to focus on relevant biological textures, such as lateral lines and body contours, rather than environmental noise.

Due to its lightweight characteristics and flexible integration, C2ICARE can be readily plugged into different computer vision architectures to enhance their discriminative power with minimal overhead. Its efficient design makes it particularly suitable for fog computing platforms deployed in underwater sensor networks, where memory bandwidth, power consumption, and computational resources are severely constrained. While the current study was specifically optimized for subaquatic environments, we believe the core principles of the operator are applicable to a broader range of monitoring tasks. Future research will focus on evaluating the module within terrestrial domains using alternative training methodologies and hyperparameter configurations to validate its generalizability across diverse physical constraints. Additionally, future efforts will transition from software simulation to physical edge-device integration on AUVs to benchmark real-time power consumption, computational latency, and memory bandwidth efficiency under strict operational hardware limits.

## Figures and Tables

**Table 1:** Computational complexity and structural scale comparison between the baseline CARE block and the proposed C2ICARE module.

Module	Input Size (H × W, C)	Parameters (M)	GFLOPs
CARE Block (Original)	20 × 20, 256	0.853	0.686
C2ICARE (Full Wrapper)	20 × 20, 256	0.207	0.166

**Table 2:** Structural characterization and partition breakdown of the dataset.

Dataset Metric	Training Subset	Validation Subset	Testing Subset	Total Dataset
Image Resolution (px)	1392 × 1040/1228 × 1027	1392 × 1040/1228 × 1027	1392 × 1040/1228 × 1027	1392 × 1040/1228 × 1027
Total Images	5652	309	918	6879
Bluewhiting instances	4424	150	470	5044
Herring instances	5116	336	933	6385
Mackerel instances	4800	165	559	5524
Mesopelagic instances	4767	152	404	5323
Total Bounding Boxes	19,107	803	2366	22,276

**Table 3:** Comparative analysis of the C2ICARE module against other attention mechanisms.

Model	mAP@0.5:0.95 (95% CI) ^1^	Parameters	T_inf_ (ms)	GFLOPs
YOLO26n + CoordAtt	0.6774 ± 0.0203	2,140,144	2.61	5.009
YOLO26n + FasterBlock	0.6912 ± 0.0218	2,427,328	2.59	5.236
YOLO26n + ImCA	0.6990 ± 0.0194	2,139,616	2.66	5.021
YOLO26n + CBAM	0.7003 ± 0.0184	2,135,860	2.91	5.008
YOLO26n + C2ICARE	0.7033 ± 0.0207	2,334,112	2.44	5.160

^1^ CI: 95% Confidence Interval computed with an expansion factor of k = 2.

**Table 4:** Multi-objective ranking and Pareto efficiency results. Averaged across three random seeds (0, 1, and 2).

Model	Score	Overfitting	Pareto Frontier
YOLO26n + C2ICARE	0.6189	−0.1584	Yes
YOLO26n + CBAM	0.6058	−0.1698	Yes
YOLO26n + ImCA	0.5956	−0.1912	Yes
YOLO26n + CoordAtt	0.5956	−0.2088	Yes
YOLO26n + FasterBlock	0.3211	−0.1655	No

**Table 5:** Detection discrepancy metrics as a function of confidence threshold (θ) for the fine-tuned YOLO26n + C2ICARE model. Values are reported as mean ± expanded uncertainty (95% CI).

Threshold (θ)	MAE (95% CI) ^1^	MAPE (95% CI)	Bias (95% CI)	$R_{count}^{2}$	Difference (∆)
0.10	1.328 ± 4.186	47.82% ± 127.94%	1.298 ± 4.231	0.0278	+1187
0.15	0.897 ± 2.895	34.12% ± 103.12%	0.812 ± 2.994	0.5418	+743
0.20	0.659 ± 2.178	25.31% ± 85.64%	0.518 ± 2.328	0.7436	+473
0.25	0.508 ± 1.699	19.61% ± 72.51%	0.289 ± 1.894	0.8451	+264
0.30	0.441 ± 1.524	17.14% ± 66.89%	0.106 ± 1.751	0.8775	+98
0.35	0.421 ± 1.521	14.81% ± 57.48%	−0.068 ± 1.739	0.8806	−62
0.40	0.438 ± 1.695	13.15% ± 50.86%	−0.234 ± 1.858	0.8562	−215
0.45	0.504 ± 1.948	14.71% ± 53.44%	−0.367 ± 2.077	0.8098	−337
0.50	0.588 ± 2.241	16.38% ± 55.55%	−0.498 ± 2.341	0.7465	−458
0.55	0.695 ± 2.522	19.28% ± 59.28%	−0.639 ± 2.596	0.6719	−588
0.60	0.809 ± 2.817	22.98% ± 64.12%	−0.776 ± 2.873	0.5825	−713

^1^ CI: 95% Confidence Interval computed with an expansion factor of k = 2.

## Data Availability

The source code of the proposed C2ICARE is openly available on Github at https://github.com/VinieLee/C2ICARE-YOLO (accessed on 1 May 2026).

## Abbreviations

The following abbreviations are used in this manuscript:

C2ICARE	Convolution to Interactive Capture and Recalibration Enhancement
YOLO	You Only Look Once
FFN	Feed-Forward Network
GELU	Gaussian Error Linear Unit
BN	Batch Normalization
GFLOPs	Giga Floating Point Operations
LPS	Latency Performance Score
GESI	GFLOPs Efficiency Score Index
PEI	Parameter Efficiency Index
CBAM	Convolutional Block Attention Module
CoordAtt	Coordinate Attention
ImCA	Improved Coordinate Attention
PConv	Partial Convolution
mAP	Mean Average Precision
MAE	Mean Absolute Error
MAPE	Mean Absolute Percentage Error
CI	Confidence Interval
